# Quantifying the Role of Longitudinal Chromatic Aberration and Age in Night Vision Disturbances

**DOI:** 10.1007/s44402-026-00131-2

**Published:** 2026-06-24

**Authors:** Laura Clavé, Maria S. Millan, Laia Salido, Miguel Faria-Ribeiro, José M. González Méijome

**Affiliations:** 1 Grupo de Óptica Aplicada y Procesado de Imagen, Departamento de Óptica y Optometría, Universitat Politècnica de Catalunya-BARCELONATECH, Terrassa, Spain; 2 Clinical and Experimental Optometry Research Laboratory (CEORLab), University of Minho, Braga, Portugal

**Keywords:** Halo, Light disturbance, Longitudinal chromatic aberration, Scattering

## Abstract

**Purpose:**

The purpose of this study was to investigate and quantify the influence of stimulus chromaticity on the perception of visual disturbances, specifically halos, under dim lighting conditions. The study also investigated age-related variations in this perception.

**Method:**

Fifty healthy participants were divided into two age groups of 25 each: young adults (<25 years) and older adults (>54 years). The halo perception was quantified using the light disturbance analyser (LDA), a validated device designed to assess visual disturbances such as halos and glare under controlled lighting conditions. To assess the effect of stimulus chromaticity on halo perception, three filters with spectral transmittances centred in the red, green and blue regions of the visible spectrum were used. Measurements were recorded both before and after compensating for longitudinal chromatic aberration (LCA).

**Results:**

In both age groups, white and green colours produced the smallest angular size of the perceived halo, followed by red, whereas blue induced the largest halo size. While LCA compensation under blue light was sufficient for the younger group to perceive a halo size similar to that under white light, this compensation proved insufficient for the older group.

**Conclusions:**

Perceived halo size was greatest when caused by a blue stimulus, followed by red light, while white and green sources yielded halos with comparable, smaller sizes across both age groups. The influence of age on perceived halo size under blue light was statistically significant. Furthermore, LCA compensation resulted in a greater benefit in perceived halo size for the younger, compared with the older group, under blue light.

Key Points

- Perceived halo size was significantly larger for blue stimuli compared to other colours (green, red), consistent with the spectral characteristics of longitudinal chromatic aberration; halo size with blue light proved to be even larger for older eyes.
- Compensation for longitudinal chromatic aberration effectively reduced perceived halo size, particularly for the large halos induced by blue light, demonstrating greater mitigation in younger subjects.
- The limited evidence regarding the effects of the aging eye can be attributed to several interacting factors: smaller pupils reducing halo size, increased lens density lowering short-wavelength transmittance and a small age gap between groups; older subjects also had good baseline visual quality, masking subtle age-related effects.

## Introduction

Evaluation of visual function often involves examining various perceptual abilities, including visual acuity, contrast sensitivity and the perception of colour, depth and movement. Other measurements of visual performance, particularly those tested under low-light conditions, are not usually included in standard clinical assessments, even though they can compromise retinal image quality and overall visual function substantially in real-world environments. Thus, certain activities such as night driving may become challenging.

In dim light, as the pupil enlarges, visual disturbances become more noticeable, particularly when the eye is exposed to intense light sources. These effects can occur even in healthy eyes due to increased sensitivity to optical imperfections and light scatter.

The assessment of light vision disturbances such as halos, glare and starbursts has become increasingly important in ophthalmology. Some ocular conditions may increase intraocular scatter and give rise to photic phenomena, including corneal irregularities [1], corneal refractive surgery [2], presbyopic correction modalities such as multifocal contact lenses or intraocular lenses [3] and age-related changes in the ocular media [4]. Traditional evaluation methods often rely on subjective patient reports or psychometric questionnaires [5, 6], as well as devices that quantify light disturbances objectively [7].

Most of the tests for night vision disturbances currently available use achromatic stimuli, despite the fact that individuals are regularly exposed to a range of coloured light sources in real-world night-time environments, such as streetlights and vehicle headlights.

The perception of chromatic stimuli, as well as intraocular scattering and straylight, is not only influenced by the eye’s differential spectral sensitivity to various wavelengths but also by chromatic aberration. Longitudinal chromatic aberration (LCA) creates a chromatic difference in power that makes an emmetropic eye function as myopic or hypermetropic, depending on the wavelength of the stimulus (see, for instance, [8–12]). In addition, age-related changes in the density of the crystalline lens can alter the transmittance of specific spectral bands, suggesting that the chromatic light disturbances experienced under low illumination, such as during night-time driving, may be influenced by the observer’s age, amongst other factors [13]. Wavelength-dependent characteristics of fundus reflectance, which are themselves associated with individual pigmentation, may also have a strong relationship with vision-impairing straylight [14]. Short wavelengths have been shown to increase halo size in young observers [15], but the effect of light stimuli chromaticity on the visual disturbances experienced by the aging eye has rarely been studied.

The aim of the present study was to investigate the effect of stimulus chromaticity on the perception of visual disturbance and its variation with age. To this end, the halo perceived by patients under dim lighting conditions was quantified using stimuli of different wavelengths, with stimulus intensity adjusted to achieve equivalent luminance based on the Commission Internationale de l’Éclairage (CIE) spectral luminous efficiency for photopic vision [16]. Previous research justified the use of this photopic efficiency standard for analysing the visual response to chromatic stimuli under dim conditions [16, 17]. The influence of LCA was accounted for in the measurement protocol, enabling the evaluation of both effects, i.e., age and LCA, separately. Results include a detailed intra- and inter-group analysis across two distinct age groups.

## Material and method

### Subjects

Fifty healthy observers were enroled in the study: a younger group of 25 young adults (age ≤ 25 years, from 18 to 25 years) with mean ± standard deviation (SD) equal to 23.09 ± 1.68 years and an older group of 25 adults (age ≥ 54 years, from 54 to 68 years, mean 57.08 ± 2.56 years). The inclusion criteria were: phakic subjects with no colour vision deficiencies, no prior refractive surgery and no media opacities or ocular pathology. All had a spherical equivalent within ±5.00 D and best-corrected visual acuity < 0.10 logMAR. All participants provided informed consent in accordance with the Declaration of Helsinki. Subjective refraction was performed in both eyes and the eye with better visual acuity was chosen for monocular examination. Pupil diameter of all participants was measured in mesopic conditions using a MYAH (Topcon.com). Table 1 shows the sociodemographic data of participants.

**Table 1:** Participant data for age (years), pupil diameter (mm) and spherical equivalent (dioptres, D) expressed as mean ± sd.

Group	Age (years)	Pupil size (mm)	Spherical equivalent (D)
Younger (≤ 25 years)	23.09 ± 1.68	6.06 ± 0.81	−0.97 ± 1.35
Older (≥ 54 years)	57.08 ± 2.56	4.58 ± 0.66	−1.34 ± 2.49

Each group had 25 participants.

### Light Disturbance Analyser (LDA)

The LDA (Binarytarget Lda, Braga, Portugal) was used to quantify the halo perceived by the patients. Its radiometric characterisation and validation were reported previously [18, 19]. The sensitivity of the LDA for evaluating optical distortion has been explored and confirmed in earlier studies [7, 20–23]. The LDA system was designed specifically to assess the effects of optical aberrations and dysphotopsia such as halos, glare and starbursts, which may be perceived under varying lighting conditions, particularly in dim or night-time settings.

The apparatus comprises a black electronic board incorporating a central high-luminance (3000 cd/m²) white light-emitting diode (LED) as the source of glare or disturbance. Surrounding this central light, 240 smaller LEDs with luminance levels of up to 6 cd/m² are arranged radially to cover a visual field of 10°, when viewed at a distance of 2 m. The spectral distribution of the central and peripheral LEDs is detailed in the supplemental file (Fig. S1). These peripheral LEDs function as probes to determine the spatial extent of glare perception across different regions of the visual field.

### Examination Procedure

To assess the effect of stimulus chromaticity on halo perception, three filters of different spectral transmittance distribution were employed, i.e., red (R) (FD1R, Thorlabs.com), green (G) (FD1G, Thorlabs.com) and blue (B) filter (FD1B, Thorlabs.com) (Figs. S2–S4 of the supplemental file) during the examination. In the initialisation of the experiment, a spectral radiometer (Spectrascan PR 715, photoresearch.com) was used to measure the resulting central LED power distribution from the LDA per each R, G, B filter spectral transmittance. This measurement was repeated with the surrounding LEDs of the LDA. The radiometric and photometric measures allowed the spectral power distributions of the resulting stimuli and their luminance to be obtained.

To equalise the luminance resulting from the combination of the central LED with each of the R, G and B filters, the following procedure was applied: first, the luminance of the central LED was measured using the blue filter at the maximum intensity provided by the device, as the blue filter provided the greatest attenuation of the three. Subsequently, to match the luminance obtained with the blue filter for the green, red and white (no filter) conditions, the LDA’s intensity was adjusted and an additional neutral density (ND) filter was applied (Absorptive ND filter NE05B, Thorlabs.com). The resulting luminance values were 200 cd/m² for the central LED and 0.45 cd/m² for the peripheral LEDs.

The examination was performed monocularly in dim conditions, with the sole illumination from the central LED of the LDA device. Observers were located 2 m in front of the LDA with the visual axis aligned with the central LED. For measurements, the best refractive correction and a +0.50 D lens (to compensate for the 2 m distance) was used throughout the assessment. The refractive correction lenses, colour and neutral filters were placed in a trial frame on the patient’s face.

The procedure was as follows: while the observer was maintaining fixation on the central LED, peripheral stimuli were presented around a central light source and the semi-meridians were explored in random order. Stimuli were displayed following an “in–out” strategy, in which radial LEDs were activated sequentially from the centre toward the periphery until the participant detected the stimulus. Upon detection, the participant pressed a response button and the system registered the result in that semi-meridian and proceeded to the next semi-meridian. The angular separation between semi-meridians was 30°.

Three measurements were obtained for each semi-meridian. The extent of light distortion along each semi-meridian was defined as the mean of these three measurements. If the standard deviation of the three measurements exceeded 20% of the mean value, then the instrument automatically repeated the measurements for that semi-meridian until the variability fell below this threshold.

The angular size of the light distortion region was derived from the best-fit circle radius (BFCRad). Since the distortion area forms an irregular polygon defined by connecting the outer limits of distortion across all semi-meridians, the circle that best fitted this shape was computed. The radius of this circle corresponds with the average distortion extent across all the semi-meridians evaluated (length), initially expressed in millimetres and subsequently converted into angular units to obtain the light distortion angular size.

The observers were instructed on the procedure and completed a preliminary trial to familiarise themselves with the visual test. The experiment included six conditions: four baseline tests, corresponding to White (W, with ND filter), Red (R, red filter plus ND filter), Green (G, green filter plus ND filter), and Blue (B, blue filter), and two additional tests in which the blur produced by the chromatic difference of refraction was compensated for, one for Green (G_LCA_) and one for Blue (B_LCA_). To estimate the lens power needed to compensate for the chromatic difference of refraction (CDRx), the following procedure was used.

Using the centre wavelength (CWL) and the full width at half maximum transmission (FWHM), the bandwidth of each R, G and B filter, measured with the spectroradiometer (Spectrascan PR 715, photoreseach.com), was derived to calculate the induced CDRx based on Applegate’s results [12]. The supplementary lens power (with ±0.25 D precision) required to compensate for each R, G and B CDRx (Table 2, top right column) was determined.

**Table 2:** Description of the filters used in the examination.

Test	Filter	ND filter	CWL (nm)	FWHM (nm)	LCA compensation^a^ (D)
White	–	Yes	–		–
Red	R	Yes	609	43.9	–
Green	G	Yes	555	72.4	−0.25
Blue	B	No	460.6	23.2	−0.75

The patient’s best distance correction was adjusted by adding +0.50 D to compensate for the chart viewing distance (2 m). Further, a supplementary lens was added to compensate for the longitudinal chromatic aberration (LCA), with ±0.25 D precision.

*CLW* centre wavelength, *FWHM* full width at half maximum transmission. *ND* neutral density.

^a^Supplementary lens power (D) to add to the subject’s refraction.

### Statistical analysis

Statistical analysis was performed using IBM SPSS software version 28.0 (ibm.com). The intra-subject effect of colour was examined using paired-samples *t*-tests for pairwise comparisons between W and colour tests. The difference between G, B and their corresponding values, adjusted for LCA, was also evaluated. Additionally, to assess the magnitude of the observed differences, the effect size was calculated and expressed as Cohen’s *d*. Effect sizes close to *d* = 0.2 indicate a small difference, while values around 0.4 and exceeding 0.8 indicate moderate and large differences, respectively.

To evaluate the interaction between condition and group, specifically, whether the pattern of differences across tests varies according to age group, independent-samples t-tests were conducted. To ensure the validity of the results, the statistical assumptions underlying the analyses were assessed, including data normality, sphericity (Mauchly’s test) and homogeneity of variances between groups (Levene’s test). If any of these assumptions were not met, then appropriate corrections were applied, such as the Greenhouse–Geisser correction for sphericity, or non-parametric methods were considered if necessary. The level of significance was set at *p* < 0.05. Bonferroni correction was not applied due to the exploratory nature of this study.

## Results

**Fig. 1:**
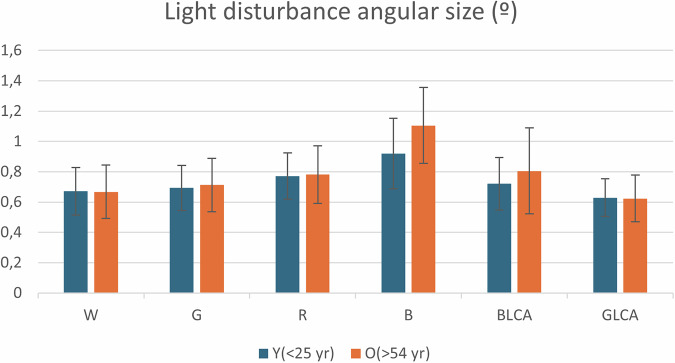
Mean angular size in degrees (°) and standard deviation of the light disturbance for the two groups of observers. Blue bars = younger (Y) group, orange bars = older (O) group. B blue, G green, LCA compensated for longitudinal chromatic aberration, R red, W white.

Figure 1 shows the mean angular size of the light disturbance (halo) for the two age groups. In both groups, pairwise comparisons yielded similar results, with statistically significant differences established between White–Red and White–Blue (Table 3). White and Green colours produced the smallest angular sizes of the perceived halo, followed by Red, whereas Blue induced the largest perceived halo size in both groups. For the LCA-compensated measures (B_LCA_, G_LCA_), a statistically significant difference was found for the W–B_LCA_ pair in the older group.

**Table 3:** Pairwise comparison for the two age groups.

	Younger (age < 25)		Older (age > 54)	
	*ρ*	Cohen’s *d*	*ρ*	Cohen’s *d*
W–G	0.14	0.22	0.2	0.16
W–R	**<0.01**	*0.58*	**0.01**	*0.46*
W–B	**<0.01**	1.23	**<0.01**	1.39
W–B_LCA_	0.47	0.32	**0.04**	*0.36*
W–G_LCA_	**0.15**	0.21	0.08	0.28
B–B_LCA_	**<0.01**	1.15	**<0.01**	1.69
G–G_LCA_	**0.02**	*0.42*	**0.02**	*0.45*

Cohen’s *d* values: Effect sizes close to 0.2 indicate a small difference (indicated as double underlined values), values around 0.4 denote a moderate difference (indicated in italic) and values exceeding 0.8 correspond to large differences (indicated as underlined values). Bold values indicate statistical significance.

*B* blue, *B*_*LCA*_ blue corrected for longitudinal chromatic aberration, *G* green, *G*_*LCA*_ green corrected for longitudinal chromatic aberration, *R* red, *W* white.

Analysis of blue and green, compared with their respective LCA compensated counterparts (B_LCA_, G_LCA_) demonstrated statistically significant differences in both age groups (Table 3).

Effect size analysis enabled evaluation of the magnitude of the observed differences between experimental conditions. For the younger group, comparison between W and R (*d* = 0.58) and G–G_LCA_ (*d* = 0.42) revealed a medium effect, while that comparison between W and B (*d* = 1.23) indicated the largest effect, demonstrating consistent and robust differences in both comparisons. Similarly, comparison between B and B_LCA_ yielded a large effect size (*d* = 1.15), reflecting a marked difference following the application of the LCA correction. In contrast, the W–G, W–B_LCA_ and W–G_LCA_ pairs showed a relatively small effect size (*d* = 0.22, *d* = 0.32 and *d* = 0.21, respectively), suggesting that no practically relevant differences were present.

The older group exhibited effect sizes similar in magnitude to those observed in the younger group. Notably, the Cohen’s *d* values for the W–B and B–B_LCA_ pairs were even higher in the older compared with the younger group, indicating a larger halo size under the B condition. The only distinction between the groups was observed in the W–B_LCA_ pair, which demonstrated a moderate effect size (*d* = 0.36) in the older group.

In the inter-group analysis, blue illumination yielded significant differences between the two groups, with an increased light disturbance in the older group. No differences were found in the angular size of the halo for the other illuminations (Table 4).

**Table 4:** Inter-group analysis for each condition.

	Inter-group analysis (*p*-value)
	*ρ*
W	0.48
G	0.33
R	0.29
B	**<0.01**
B_LCA_	0.12
G_LCA_	0.35

*ρ* independent sample *T*-test, *B* blue, *B*_*LCA*_ blue corrected for longitudinal chromatic aberration, *G* green, *G*_*LCA*_ green corrected for longitudinal chromatic aberration, *R* red, *W* white. Bold values indicate statistical significance.

## Discussion

The findings of this study demonstrate that the halo size was greatest when caused by a blue stimulus (*p* < 0.01 in Table 3), followed by red light (*p* = 0.01), while white and green sources yielded halos with comparable smaller sizes across both age groups. This pattern aligns with results by Castro-Torres et al. [15], who also observed the largest halo sizes under blue light stimulation in young observers, despite using a different methodological approach.

The current results are consistent with the spectral dependence of LCA. Following LCA compensation, using −0.25 D for green light and −0.75 D for blue light, both age groups experienced statistically significant differences in perceived halo size (Table 3). Specifically, under green and blue light, the difference was significant for both age groups (*p* = 0.02 and <0.01, respectively). As noted previously, Bonferroni correction was not applied due to the exploratory nature of this study; however, it should be noted that the W–B and B–B_LCA_ comparisons in both age groups (Table 3) would remain significant even under this conservative adjustment.

Intraocular light scattering may be an additional contributing factor. The pronounced perception of halos under blue light might be attributed, in part, to the Rayleigh-type wavelength dependence (*λ*^−4^), where ocular straylight would be highest for short wavelengths and lowest for medium wavelengths. However, Coppens et al. [24] demonstrated that intraocular straylight does not strictly adhere to the *λ*^−4^ Rayleigh pattern, being dependent additionally on ocular pigmentation and age. While age-related changes increase straylight across all wavelengths, the reduced pigmentation of Caucasian eyes (a common feature of both of the age groups) opposed the Rayleigh pattern by producing greater straylight in the long-wavelength range. The results revealed the influence of the aging eye on perceived halo size under blue light, showing a statistically significant difference in the inter-group analysis (*p* < 0.01 in Table 4). Furthermore, while the LCA compensation under blue light was sufficient for the younger group, resulting in a halo size similar to that under white light (*p* = 0.47 and low effect size *d* = 0.32), this compensation proved insufficient for the older group. The older group maintained a statistically significant difference between the halo size perceived under white and LCA-compensated blue light (*p* = 0.04, *d* = 0.36 -Table 3).

Further, inter-group comparisons between both age groups (Table 4) did not reveal statistically significant differences in perceived halo size when subjects were exposed to white, red and green stimuli. This lack of significance also held true for LCA-compensated vision under green and blue light. This limited evidence regarding the effects of the aging eye can be attributed to several factors:

- *Pupil diameter*: In this experiment, pupil diameter was larger in the younger than in the older group (6.06 ± 0.81 vs. 4.58 ± 0.66, *p* = 0.02). The larger pupil of the younger group would have increased the impact of visual disturbance, making it comparable to that noticed by the older group. An association between pupil diameter and halo size was demonstrated by Zhao et al. [25], who reported larger haloes in those observers with greater pupillary diameter under light stimulation. The impact of aberrations is also dependent on pupil size, thereby affecting the younger group here.
- The differences in age between the two groups examined here may not be sufficiently large. For instance, although Puell et al. [26] used a different methodology and achromatic stimuli, they reported that halo size did not increase significantly with age until approximately 50–59 years. The mean age of the older group here (57.08 ± 2.56 yr) fell within this age interval; thus, only contributing marginally to an increase in halo size. Furthermore, the present inclusion criteria minimised the likelihood of media opacities, as subjects were required to have healthy eyes and a best-corrected distance visual acuity < 0.10 logMAR. In fact, all older group participants achieved 0.00 logMAR.
- *Lens density*: This increases with age, leading to decreased transmittance, particularly at short wavelengths [27, 28]. This reduction in transmittance may partially counteract the age-related increase in straylight, which could account for the similar halo sizes observed in the two age groups for red and green colours. Glare and halo testing have important implications in ophthalmology (e.g., with cataract and refractive surgery) but still involve many issues [29]. This study did have some limitations. First, due to the high attenuation of the blue filter, the test was conducted under relatively low luminance conditions (200 cd/m²) compared with the levels typically employed in light disturbance examinations (up to 3000 cd/m²). This factor may have masked potentially larger halo sizes in the older cohort. As noted previously, the differences in mean pupil size and the relatively small age gap between the groups might also have influenced the halo sizes observed. However, performing the test under physiological conditions with a natural pupil size offers valuable insight into how subjects of different ages perceive chromatic stimuli in a real-world context.

## Supplementary information

Supplementary File

## Data Availability

The anonymised data supporting the findings of this study are available within the paper and its Supplementary Information (Supplementary Tables 1 and 2).
