# Supplementary material for: Quantifying the Role of Longitudinal Chromatic Aberration and Age in Night Vision Disturbances
Source: Ophthalmic Physiol Opt. 2026 Jun 24;46(4):946–51. doi: 10.1007/s44402-026-00131-2 (PMC13395955; doi:10.1007/s44402-026-00131-2)
Supplement: Supplementary file 1 — Supplementary File [file 44402_2026_131_MOESM1_ESM.docx]

**Supplementary file**

Dichroic filters transmission (0º angle incidence)

**Fig S1** : Transmission (%) spectrum of the FD1R red dichroic filter at 0° angle of incidence. The filter shows high transmission (>90%) for wavelengths above 550 nm and blocks shorter wavelengths

**Fig S2**: Transmission (%) spectrum of the FD1G green dichroic filter at 0° angle of incidence. The filter exhibits peak transmission between 500–650 nm with high rejection outside this range

**Fig S3**: Transmission (%) spectrum of the FD1B blue dichroic filter at 0° angle of incidence. The filter shows high transmission (<500 nm) with sharp cutoff above 500 nm.

**Fig S4**: Mean irradiance of the central and peripheral LEDs of the Light Disturbance Analyzer

Table 1. Halo angular size of each participant under conditions W, G, R, B_LCA and G_LCA. Young group (age under 25 years).

| ID | W | G | R | B | B_LCA | G_LCA |
| --- | --- | --- | --- | --- | --- | --- |
|  |  |  |  |  |  |  |
| 1 | 0,69 | 0,53 | 0,61 | 0,92 | 0,61 | 0,50 |
| 2 | 0,53 | 0,61 | 0,80 | 0,73 | 0,53 | 0,50 |
| 3 | 0,88 | 0,88 | 0,96 | 1,03 | 0,92 | 0,50 |
| 4 | 0,53 | 0,50 | 0,65 | 0,84 | 0,46 | 0,65 |
| 5 | 0,57 | 0,53 | 0,88 | 0,92 | 0,46 | 0,57 |
| 6 | 0,80 | 0,92 | 0,84 | 0,92 | 0,92 | 0,92 |
| 7 | 0,92 | 0,92 | 0,96 | 0,92 | 0,92 | 0,84 |
| 8 | 1,07 | 0,96 | 1,07 | 1,60 | 1,07 | 0,84 |
| 9 | 0,84 | 0,76 | 0,92 | 0,88 | 0,53 | 0,46 |
| 10 | 0,46 | 0,50 | 1,03 | 0,73 | 0,53 | 0,57 |
| 11 | 0,65 | 0,69 | 0,65 | 0,99 | 0,69 | 0,73 |
| 12 | 0,50 | 0,88 | 0,69 | 1,19 | 0,92 | 0,61 |
| 13 | 0,88 | 0,73 | 0,73 | 0,84 | 0,61 | 0,46 |
| 14 | 0,53 | 0,46 | 0,46 | 0,50 | 0,46 | 0,50 |
| 15 | 0,57 | 0,53 | 0,73 | 0,61 | 0,61 | 0,61 |
| 16 | 0,61 | 0,76 | 0,53 | 1,41 | 0,80 | 0,57 |
| 17 | 0,57 | 0,61 | 0,69 | 0,84 | 0,73 | 0,69 |
| 18 | 0,65 | 0,69 | 0,76 | 0,88 | 0,80 | 0,65 |
| 19 | 0,61 | 0,65 | 0,69 | 0,92 | 0,84 | 0,73 |
| 20 | 0,69 | 0,73 | 0,80 | 0,92 | 0,80 | 0,57 |
| 21 | 0,53 | 0,57 | 0,65 | 0,76 | 0,73 | 0,61 |
| 22 | 0,69 | 0,69 | 0,73 | 0,92 | 0,80 | 0,62 |
| 23 | 0,46 | 0,50 | 1,02 | 0,72 | 0,53 | 0,57 |
| 24 | 0,80 | 0,92 | 0,85 | 0,92 | 0,92 | 0,92 |
| 25 | 0,61 | 0,75 | 0,53 | 1,08 | 0,80 | 0,57 |

Table 2. Halo angular size of each participant under conditions W, G, R, B_LCA and G_LCA. Mature group (age over 54 years).

| ID | W | G | R | B | B_LCA | G_LCA |
| --- | --- | --- | --- | --- | --- | --- |
|  |  |  |  |  |  |  |
| 1 | 0,50 | 0,80 | 0,92 | 0,99 | 0,73 | 0,50 |
| 2 | 0,57 | 0,53 | 0,50 | 0,99 | 0,57 | 0,61 |
| 3 | 0,46 | 0,61 | 0,61 | 0,88 | 0,50 | 0,53 |
| 4 | 0,57 | 0,53 | 0,61 | 1,26 | 0,99 | 0,57 |
| 5 | 0,57 | 0,61 | 0,69 | 1,03 | 1,07 | 0,76 |
| 6 | 0,61 | 0,61 | 0,73 | 1,22 | 0,69 | 0,50 |
| 7 | 0,61 | 0,69 | 0,92 | 1,11 | 0,96 | 0,53 |
| 8 | 0,80 | 0,88 | 0,88 | 0,88 | 0,73 | 0,61 |
| 9 | 0,88 | 0,57 | 0,61 | 0,96 | 0,61 | 0,50 |
| 10 | 0,69 | 0,76 | 0,50 | 1,49 | 1,19 | 0,88 |
| 11 | 0,53 | 0,80 | 0,76 | 1,49 | 1,60 | 0,84 |
| 12 | 0,92 | 0,65 | 0,69 | 1,37 | 0,92 | 0,61 |
| 13 | 1,11 | 0,53 | 0,69 | 0,96 | 0,61 | 0,96 |
| 14 | 0,61 | 0,92 | 0,88 | 1,79 | 1,30 | 0,50 |
| 15 | 0,57 | 1,11 | 1,15 | 0,88 | 0,53 | 0,57 |
| 16 | 0,99 | 1,03 | 1,15 | 0,92 | 0,50 | 0,96 |
| 17 | 0,46 | 0,88 | 1,07 | 0,96 | 0,99 | 0,50 |
| 18 | 0,61 | 0,65 | 0,96 | 0,92 | 0,61 | 0,50 |
| 19 | 0,92 | 0,78 | 0,69 | 0,99 | 0,57 | 0,50 |
| 20 | 0,61 | 0,50 | 1,03 | 0,84 | 0,66 | 0,57 |
| 21 | 0,57 | 0,61 | 0,65 | 1,30 | 0,69 | 0,62 |
| 22 | 0,50 | 0,96 | 0,92 | 1,11 | 0,80 | 0,62 |
| 23 | 0,69 | 0,57 | 0,99 | 1,11 | 0,90 | 0,62 |
| 24 | 0,88 | 0,57 | 0,61 | 1,10 | 0,61 | 0,50 |
| 25 | 0,46 | 0,61 | 0,70 | 0,88 | 0,55 | 0,53 |
